# Truncated VZV gE Induces High-Titer Neutralizing Antibodies in Mice

**DOI:** 10.3390/vaccines12101139

**Published:** 2024-10-04

**Authors:** Jiehui Wu, Hai Li, Yanping Yuan, Ruichen Wang, Tianxin Shi, Ziyi Li, Qianqian Cui, Shihong Fu, Kai Nie, Fan Li, Qikai Yin, Jiayi Du, Huanyu Wang, Songtao Xu

**Affiliations:** 1 National Key Laboratory of Intelligent Tracking and Forecasting for Infectious Diseases, National Institute for Viral Disease Control and Prevention, Chinese Center for Disease Control and Prevention, Beijing 102206, China; wujiehui5439@163.com (J.W.); yuanyanping_love@126.com (Y.Y.); wangrc96@163.com (R.W.); shitianxin2022@163.com (T.S.); liziyi_99@163.com (Z.L.); cuiqq@ivdc.chinacdc.cn (Q.C.); fush@ivdc.chinacdc.cn (S.F.); niekai@ivdc.chinacdc.cn (K.N.); lifan@ivdc.chinacdc.cn (F.L.); yinqk@ivdc.chinacdc.cn (Q.Y.); wanghy@ivdc.chinacdc.cn (H.W.); 2 WHO WPRO Regional Reference Measles/Rubella Laboratory, National Institute for Viral Disease Control and Prevention, Chinese Center for Disease Control and Prevention, Beijing 102206, China; lihai@ivdc.chinacdc.cn; 3 Yale School of Public Health, New Haven, CT 06510, USA; jiayidu1028@gmail.com

**Keywords:** varicella zoster virus, herpes zoster, glycoprotein E, vaccine, neutralizing antibody

## Abstract

**Backgrounds:** A contemporary public health challenge is the increase in the prevalence rates of herpes zoster (HZ) worldwide. **Methods:** In this work, the gE gene structure was analyzed using bioinformatics techniques, and three plasmids of varying lengths, tgE537, tgE200, and tgE350, were expressed in Chinese hamster ovary (CHO) cells. These proteins were used to immunize BALB/c mice with Al/CpG adjuvant; ELISPOT and FCM were used to evaluate cellular immunity; and ELISA, VZV microneutralization, and FAMA assays were performed to detect antibody titers. **Results:** Target protein concentrations of 1.8 mg/mL for tgE537, 0.15 mg/mL for tgE200 and 0.65 mg/mL for tgE350 were effectively produced. The ability of the three protein segments to stimulate CD4^+^ and CD8^+^ T cells, as well as to cause lymphocytes to secrete IFN-γ and IL-4, did not significantly differ from one another. Both tgE537 and tgE350 were capable of generating VZV-specific antibodies and neutralizing antibodies, while tgE350 had the highest neutralizing antibody titer (4388). There was no equivalent humoral immune response induced by tgE200. **Conclusions**: The results of this investigation provide the groundwork for the creation of HZ recombinant vaccines using truncated proteins as antigens.

## 1. Introduction

A class of encapsulated viruses having double-stranded DNA genomes is known as herpes viruses. Human herpes viruses are divided into three subfamilies, alpha, beta, and gamma, based on variations in their genomic sequences, structures, and physicochemical characteristics. Humans are the only host of the varicella zoster virus (VZV), which is a member of the alpha herpesvirus subfamily and consists of a single serotype. Varicella, or chickenpox, is the result of a primary VZV infection that strikes young people by the inhalation of infectious droplets or contact with contaminated materials from skin sores. Vesicle-borne viruses enter sensory neurons at the dermoepidermal junction and proceed retrogradely to the sensory ganglia. Alternatively, they enter neurons by viremia and finally become latent within the cranial nerve ganglia or dorsal root ganglia [1]. Immunocompromised or immunodeficient states can cause the reactivation of VZV, leading to ganglion infection (ganglionitis) and resulting in typical neuropathic pain [2,3]; subsequently, VZV is transported along microtubules within sensory axons to infect epithelial cells, thus causing a rash within the dermatome governed by a single sensory neuron [4], known as herpes zoster (HZ), which is a secondary infection caused by VZV.

Despite the extensive documentation of the worldwide occurrence of herpes zoster (HZ), only a limited number of nations and areas have carried out statistical analyses and epidemiological studies. The incidence of HZ increased from 286 to 579.6 cases per 100,000 person-years, with an average yearly rise of 3.1%, according to U.S. cohort research (1994–2018) [5]. In the Asia–Pacific area as a whole, the incidence rate of shingles is between 3 and 10 cases per 1000 people [6]. In China, the incidence rate of HZ is 4.28 per 1000 person-years overall, rising to 11.69 per 1000 person-years in the over-60 age group [7]. Postherpetic neuralgia (PHN) affects 29.8% of patients [8]. The global surge in HZ cases during the COVID-19 pandemic emphasizes the disease’s economic and social cost and the necessity of more attention. As of right now, immunization is the most effective way to avoid HZ. The vaccines that are now on the market mostly consist of recombinant protein and live attenuated vaccines. In 2019, China approved GSK’s HZ recombinant protein vaccine Shingrix, which combines the adjuvant AS01_B_ with the VZV glycoprotein E (gE) extracellular domain as the antigen [9]. VZV gE is a type I membrane protein with 623 amino acids (AAs) that is encoded by ORF 68, which is found in the unique short region of the genome. The N-terminus of the protein consists of a hydrophilic extracellular domain (AAs 1–544) with a signal peptide (AAs 1–37), a 17 AA transmembrane hydrophobic region, and a 62 AA cytoplasmic domain [10]. The most prevalent glycoprotein in infected cells, VZV gE is an essential part of the viral envelope, is essential for viral replication, and triggers humoral and cellular immune responses [11,12]. In combination with the viral kinases ORF47, ORF66, and gI, its distinct amino terminus allows it to infiltrate T cells [1]. VZV gE is a transmembrane protein that makes it a special target for antiviral responses in B-cell responses and a crucial target for neutralizing antibodies. As an antibody target, it takes part in both intercellular viral spread and antibody-dependent cellular cytotoxicity (ADCC) reactions [13].

**Figure 1:**
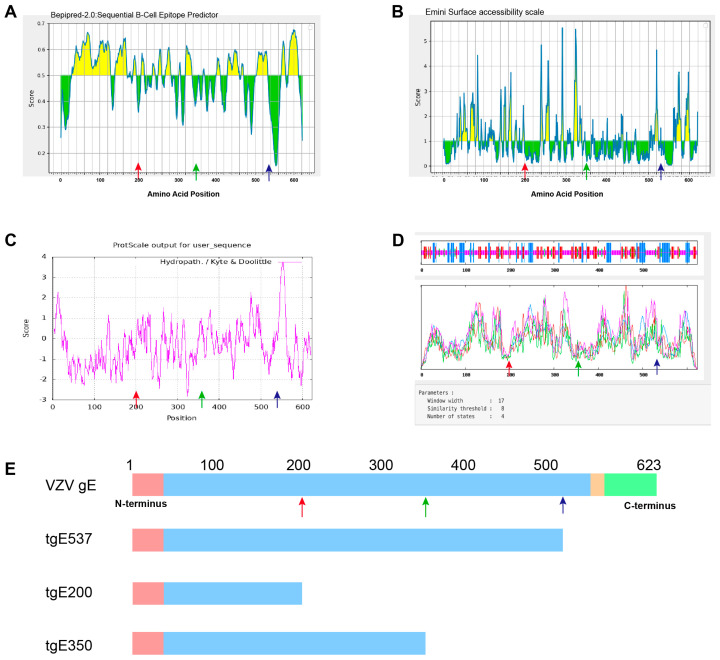
Schematics of truncated proteins and predictions from the bioinformatics analysis. (**A**) IEDB-based B-cell epitope prediction for VZV gE. (**B**) Emini Surface accessibility scale for VZV gE. (**C**) Protscale hydrophobicity prediction of gE. (**D**) VZV gE secondary structure prediction. (**E**) Schematic of VZV and the truncated proteins: tgE537 (1–537), tgE200 (1–200) and tgE350 (1–350). Color code: pink—signal peptide, light blue—extracellular domain, orange—transmembrane domain, green—cytoplasmic domain. Red arrow: position 200, green arrow: position 350, blue arrow: position 537.

This work generated truncated protein variants of AAs 1–200 and AAs 1–350 using the commercial vaccine gE antigen (AAs 1–537) as a reference, based on the structural properties of VZV gE and a related bioinformatics analysis (Figure 1). CHO cell lines were used to produce three different proteins, tgE537 (AAs 1–537), tgE200 (AAs 1–200), and tgE350 (AAs 1–350), which were then mixed with an adjuvant called Al/CpG and injected intramuscularly into BALB/c mice. This work provides experimental data and insights for the development of recombinant HZ vaccines employing shortened protein antigens by evaluating the humoral and cellular immune responses to various lengths of the E protein in mice.

## 2. Materials and Methods

### 2.1. Cells and Animals

Chinese hamster ovary cells (CHO cells) are preserved at the Arbovirus Laboratory, Institute for Viral Disease Control and Prevention, Chinese Center for Disease Control and Prevention; female BALB/c mice (aged 6–8 weeks) were purchased from Beijing Vital River Laboratory Animal Technology Co., Ltd. (Beijing, China).

### 2.2. Protein Design Methodology

The alignment of 285 VZV gE sequences from the NCBI database revealed that the gE sequence is highly conserved. Referencing human alphaherpesvirus 3 (GenBank accession number: NC_001348.1(115808..117721)), we predicted the physicochemical properties, antigenic epitopes, and secondary structure of VZV gE using tools such as IEDB Analysis Resource v2.27 (https://www.iedb.org/, accessed on 4 December 2023), Expasy 3.0 (https://www.expasy.org/, accessed on 5 December 2023), and SOPMA (https://npsa-prabi.ibcp.fr/cgi-bin/npsa_automat.pl?page=npsa_sopma.html, accessed on 16 December 2023, last tool modification: 15 March 2021).

Based on the results of the aforementioned analyses, gE was divided into three segments: AAs 1–200, AAs 1–350 and AAs 1–537. Each segment was modified to include a His tag at the C-terminus. Using BsmBI and XbaI as restriction sites, these segments were cloned into the pcDNA3.4 vector, resulting in constructs named pcDNA3.4-VZV-gE_1_–_200_, pcDNA3.4-VZV-gE_1_–_350_, and pcDNA3.4-VZV-gE_1_–_537_. The proteins expressed from these constructs were referred to as tgE200, tgE350 and tgE537, respectively, and were synthesized by General Biosystems (Anhui) Co., Ltd. (Anhui, China).

### 2.3. Recombinant Plasmid Extraction, Transfection, and Purification of Proteins

The recombinant plasmids were transformed into the *Escherichia coli* DH5-α strain and inoculated into LB medium with a final ampicillin concentration of 100 μg/mL. The culture was incubated at 37 °C until reaching an A600 of 0.6, then plasmids was extracted using QIAGEN plasmid Maxi Kit (QIAGEN, cat. #12163, Hilden, Germany). The recombinant plasmids were transfected into CHO cells using a cell transfection kit (Thermo Fisher, cat. #A29129, Waltham, MA, USA), with daily sampling to monitor the cell density and viability. The culture was terminated when cell viability fell below 80%, and the supernatant was collected after centrifugation. The proteins were purified by Ni-NTA affinity chromatography, and their concentrations were determined using a BCA assay (NCM Biotech, cat. #WB6501, Suzhou, China).

### 2.4. Western Blot

Purified tgE537, tgE200, and tgE350 were mixed with 5× protein loading buffer and boiled in a metal bath for 10 min. The samples were then subjected to electrophoresis on a 20% SDS-PAGE gel (GenScript, cat. #M00655, Nanjing, China) at 110 V for 60 min. The proteins were transferred onto a nitrocellulose (NC) membrane (Cytiva, cat. #10600002, Uppsala, Sweden) using a Tanon VE-386 transfer system. The membrane was blocked with 5% skim milk at room temperature for 2 h. An VZV gE monoclonal antibody (1:2000, ABMAX, cat. #05-0159, Beijing, China) was used as the primary antibody and incubated with the membrane for 2 h, followed by washing. HRP-conjugated goat anti-mouse IgG (1:5000, ZSGB-BIO, cat. #2B-2305, Beijing, China) was used as the secondary antibody and incubated with the membrane for 1 h, followed by washing. A high-sensitivity ECL substrate solution kit (Thermo Fisher, cat. #32209, Waltham, MA, USA) was added, and the signal was visualized using a chemiluminescence imaging system.

### 2.5. Animal Immunization

Specific pathogen-free BALB/c female mice aged 6–8 weeks and weighing 16 to 18 g were used. All mice were randomly divided into four groups (N = 6): 3 groups of subunit vaccine-immunized mice and 1 group of PBS-immunized mice as a negative control (Table 1). The mice were injected intramuscularly near the tibia. Vaccines were administered on days 0 and 21 for a total of 2 injections. Blood samples were taken on the 14th day following the administration of each vaccine. From the first vaccination, the collection days were 14 and 35. The serum was separated from the samples by centrifugation at 2000× *g* rpm for 40 min. Mouse spleens were taken two weeks after the final immunization and processed to produce a single-cell suspension for the Enzyme-Linked ImmunoSpot (ELISPOT) test and flow cytometry (FCM) analysis.

### 2.6. CMI Measurement Assays

The CMI response was measured using ELISPOT or flow cytometry assays.

For ELISPOT, spleens were collected from immunized mice, and single-cell suspensions were prepared. Splenocytes (5 × 10^5^ cells/well) were seeded into the wells of anti-mouse IFN-γ (Mabtech, cat. #3321-4HST-2, Nacka, Sweden) or IL-4 (Mabtech, cat. #3311-4HPW-2, Nacka, Sweden) antibody-precoated ELISPOT plates, followed by the addition of 10 μL of gE (5 μg/mL), the positive control (PMA + Ionomycin), or 1640 medium. The detection procedure was conducted according to the manufacturer’s instructions. Spots were counted and analyzed using a Mabtech IRIS FluoroSpot/ELISpot reader.

For the flow cytometry analysis, splenocytes (100 μL) were stained with CD45 APC-CY7 (BioLegend, cat. #103116, San Diego, CA, USA), CD3 PerCP (BioLegend, cat. #100218, San Diego, CA, USA), CD4 APC (BioLegend, cat. #100412, San Diego, CA, USA), CD8 PE-CY7 (BioLegend, cat. #100722, San Diego, CA, USA), CD45/B220 FITC (BioLegend, cat. #103205, San Diego, CA, USA), and CD49b PE (BioLegend, cat. #103506, San Diego, CA, USA) antibodies as per the recommended protocol and incubated in the dark for 15 min. After adding 1 mL of lysis buffer, the splenocytes were vortexed and incubated in the dark for 10 min, followed by centrifugation at 1500× *g* rpm for 15 min. The supernatant was discarded. The cells were washed with 1 mL of PBS, centrifuged again for 15 min, and the supernatant was discarded. The splenocytes were resuspended in 500 μL of PBS and analyzed using a BD FACSCanto II flow cytometer. Data analysis was performed with FACS Diva 8.0 software.

### 2.7. ELISA

ELISA was performed to determine the antigenicity of truncated proteins or sera antibody binding titers. The wells of 96-well microplates were coated with tgE537 (20 ng per well) overnight at 4 °C. The plates were blocked with 3% BSA in PBS for 2 h. The wells were incubated with 5-fold serum dilutions (the first dilution was 100-fold) for 1 h at 37 °C. After 5 washes with PBST, the plates were incubated with HRP-conjugated goat anti-mouse IgG (1:5000, ZSGB-BIO, cat. #2B-2305, Beijing, China), IgG1 (1:2000, APPLYGEN, cat. #C2221, China) and IgG2a (1:2000, APPLYGEN, cat. #C2223, Beijing, China) secondary antibodies for 1 h at 37 °C. The plates were washed 5 times and then incubated with 100 μL of tetramethylbenzidine (TMB) substrate (Solarbio, cat. #PR1200, Beijing, China) for color development at 37 °C for 10 min, followed by the addition of 50 μL/well of sulfuric acid to stop the reaction. Finally, the absorbance was measured at 450 nm.

### 2.8. In Vitro Microneutralization Assay

Vazyme Testing Technology Co., Ltd. (Nanjing, China) was contracted to perform a microneutralization assay. MRC-5 cells were adjusted to the proper density and seeded onto 96-well plates before being incubated overnight at 37 °C with 5% CO_2_. Serum samples were heat-inactivated in a water bath at 56 °C for 30 min. The serum was originally diluted 30-fold, followed by three-fold repeated dilutions, yielding eight dilution levels (including the initial dilution), each with two repetitions. The virus was diluted appropriately. The diluted virus was applied to both sample and viral control wells, and then neutralized for about an hour in a 37 °C, 5% CO_2_ incubator. The virus–serum neutralization products, positive controls, and back-titration samples were added to the prepared cell monolayers at 50 μL per well in duplicate. After 2 h of incubation at 37 °C with 5% CO_2_, the media were replaced with 100 μL of new culture medium in each well. The incubation period lasted around 48 h. The supernatant was then removed, the cells were fixed, and detection antibodies with fluorescent labels were added. Plates were examined using CTL equipment to quantify the infected cells. The neutralizing activity of each serum sample against VZV was determined by comparing the number of virus-infected cells in the presence of serum to that in the virus control group, and was expressed as ID50 (serum dilution inhibiting 50% of VZV infection). The proportion of infected cells at each dilution was compared to the virus control. The Reed–Muench formula was used to compute the antibody titer, with a cut off of ≥30 signifying a positive response.

### 2.9. FAMA

The FAMA test was carried out according to the Yun X protocol, with slight changes [14]. Antigen slides were prepared using VZV-infected cells as the antigen. Serum was applied to the slides (20 μL/well) after being serially diluted 1:2 with PBS. The slides were incubated for one hour at 37 °C in a humid chamber; subsequently, they were washed three times with PBS (five min each wash) and then air-dried. Following a 1 h incubation period at 37 °C in a humid environment, 10 μL/well of FITC-goat anti-mouse IgG mixed 1:200 with a 0.01% Evans blue dye solution was applied to the slides, which were subsequently cleaned with PBS, as previously indicated. An inverted fluorescence microscope was used for the observation. On the cell surface, positivity was shown by ring-shaped fluorescence, whereas negativity was indicated by the lack of the whole fluorescent ring or by the presence of just red nuclei.

### 2.10. Statistical Analysis

GraphPad Prism 9 was used to analyze the ELISA data (EC50 calculations) and to perform the statistical analysis. Differences between experimental groups were analyzed using one-way ANOVA, and group means were compared using Tukey’s multiple comparison test. *P* values in each group are indicated as * *p* < 0.05; ** *p* < 0.01; *** *p* < 0.001; **** *p* < 0.0001; and ns, not significant, *p* > 0.05.

## 3. Results

### 3.1. Characterization of Designed Proteins

The IEDB tool was used to predict potential epitopes on the gE protein surface. Potential antigenic sites within the first 350 amino acids of the protein were identified through an analysis of putative B-cell epitopes (Bepipred 2.0) and antigen surface accessibility (Emini Surface Accessibility Prediction), with particularly strong signals in the regions spanning amino acids 29–129 and 140–171 (Figure 1A,B). Bioinformatics analysis using the Protscale hydrophobicity tool from Expasy (Figure 1C) revealed highly hydrophobic sections at both the N-terminus (AAs 0–22) and C-terminus (AAs 542–562) of gE. These regions correspond to the signal peptide and the transmembrane domain, respectively. Based on previous studies from our laboratory, complemented by a secondary structure prediction using the SOPMA (Self-Optimized Prediction Method with Alignment) tool and a literature review, truncation sites were selected at the 200th and 350th amino acids to ensure the integrity of the secondary structure (Figure 1D). The shortened proteins, tgE537, tgE200 and tgE350, are shown in Figure 1E.

### 3.2. Expression, Purification, and Identification of Truncated Proteins

**Figure 2:**
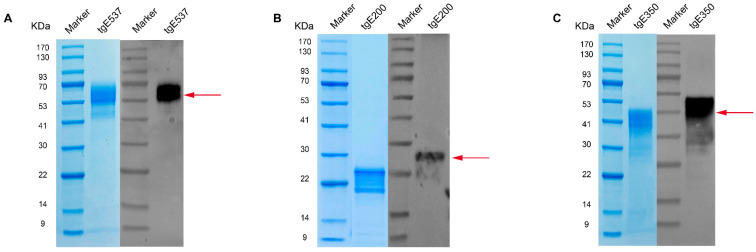
SDS-PAGE and Western blot analyses of the truncated proteins (**A**) tgE537, (**B**) tgE200, and (**C**) tgE350. SDS-PAGE (blue lanes) was used to visualize the expressed proteins and estimate their molecular weights. Western blot analysis (black lanes) using a commercial VZV gE monoclonal antibody confirmed the identity and specificity of the expressed proteins. Red arrows indicate the specific bands of purified truncated gE proteins: tgE537 (~70 kDa), tgE200 (~25 kDa), and tgE350 (~45 kDa).

CHO cells were transfected using plasmids pcDNA3.4-VZV-gE_1_–_200_, pcDNA3.4-VZV-gE_1_–_350_ and pcDNA3.4-VZV-gE_1_–_537_. Supernatants were recovered after the batch culture was fed in a shaking flask for six days. To acquire the target proteins, the supernatants were purified using Ni-NTA affinity chromatography and eluted with 300 mM imidazole. With purities over 90%, the quantities of tgE537, tgE200 and tgE350 were, respectively, 1.8 mg/mL, 0.15 mg/mL, and 0.65 mg/mL. Figure 2 displays both SDS-PAGE and Western blot results for the purified truncated gE proteins. Panels A, B, and C show the results for tgE537, tgE200, and tgE350, respectively. In each panel, the blue lane shows the Coomassie blue-stained SDS-PAGE gel, while the black lane presents the Western blot results using monoclonal antibodies against VZV gE. Red arrows indicate distinct bands at approximately 70 kDa (tgE537), 25 kDa (tgE200), and 45 kDa (tgE350). The observed molecular weights were slightly higher than the theoretical masses calculated from the amino acid sequences, consistent with the expected effect of post-translational modifications, particularly glycosylation. The specific bands detected by Western blot validate both the expression of the target proteins and their recognition by VZV gE-specific antibodies.

### 3.3. Truncated Proteins Induced Similar Cellular Immune Responses

**Figure 3:**
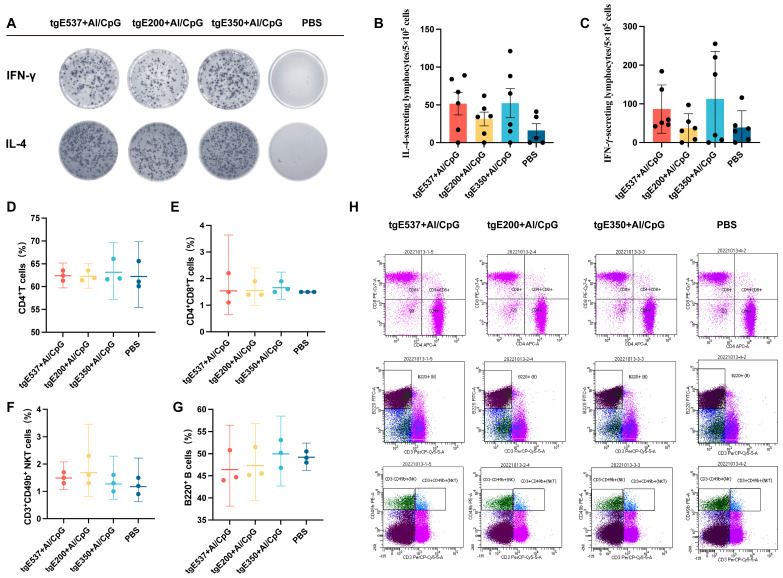
(**A**) Representative pictures of splenocytes that produce IL-4 and IFN-γ in the enzyme-linked immunospot (ELISPOT) test. (**B**,**C**) ELISPOT was used to identify the IFN-γ and IL-4 that splenocytes released after being stimulated with 10 μg/mL tgE537. N = 6 (**B**,**C**), where a single mouse is represented by each dot. (**D**–**G**) The percentages of CD4^+^ T cells, CD4^+^CD8^+^ T cells, NKT cells and B cells in splenocytes. (**H**) Pseudocolor pictures showing typical outcomes for gated CD4^+^ T cells, CD4^+^CD8^+^ T cells, NKT cells and B cells that are close to the average value. N = 3 (**D**–**G**), where a single mouse is represented by each dot. Tukey’s multiple comparisons test was used after one-way analysis of variance (ANOVA) to compare the means of each group with each other.

Mice splenocytes were obtained for the cytokine analysis two weeks after the second immunization to assess the degree of the vaccination-induced cell-mediated immune response. As demonstrated in Figure 3B, after tgE537 stimulation, there was no statistically significant difference in the number of IL-4 secreting lymphocytes generated by the tgE537 + Al/CpG and tgE200 + Al/CpG groups (the average number for tgE537 + Al/CpG was 52/5 × 10^5^ lymphocytes, and for tgE200 + Al/CpG it was 31/5 × 10^5^ lymphocytes, *p* = 0.73). The average number of lymphocytes in the tgE537 + Al/CpG and tgE350 + Al/CpG groups was 52/5 × 10^5^. The PBS group’s average number of lymphocytes was 16/5 × 10^5^, which did not differ substantially from any of the vaccinated groups. The data shown in Figure 3C indicate that there was no significant difference in the number of IFN-γ secreting cells per 5 × 10^5^ lymphocytes among the tgE537 + Al/CpG, tgE200 + Al/CpG, tgE350 + Al/CpG and PBS groups, which were, respectively, 87/5 × 10^5^, 38/5 × 10^5^, 113/5 × 10^5^, and 39/5 × 10^5^.

To analyze T cell phenotypes and cytokine secretion levels, we collected mouse splenocytes 14 days after the second immunization and performed a flow cytometry analysis (Figure 3D–H). Similar proportions of CD4^+^ T cells (Figure 3D) were generated by the tgE200 + Al/CpG and tgE350 + Al/CpG groups compared to the tgE537 + Al/CpG group. No statistically significant differences were observed among the experimental groups in the proportions of CD4^+^CD8^+^ double-positive T cells (Figure 3E), CD3^+^CD49b^+^ NKT cells (Figure 3F), and B220^+^ B cells (Figure 3G).

Despite the lack of statistical significance when compared to the PBS-injected control, the overall trend of the results seems to indicate that most of the truncated proteins are able to induce both Th1 and Th2 cell immunity in mice in the presence of adjuvant.

### 3.4. The tgE350 + Al/CpG Treatment Induced High-Titer Neutralizing Antibodies in Mice

**Figure 4:**
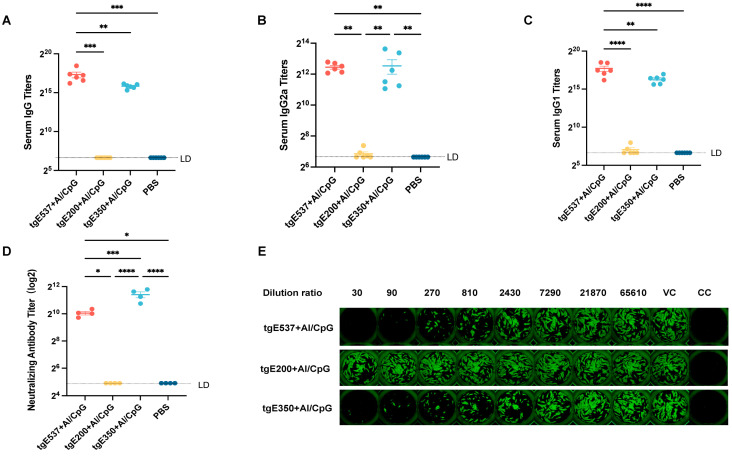
In BALB/c mice, the immunogenicity of tgE537, tgE200, and tgE350 was assessed two weeks following the two-dose vaccination. (**A**–**C**) ELISA was used to measure gE-specific IgG, IgG2a, and IgG1 titers induced by various immunological strategies, titers in (**A**–**C**) are shown in log2. (**D**) The titers of neutralizing antibodies for every group in the microneutralization experiment. (**E**) Illustrative images of neutralizing antibody titers. VC: virus control; CC: cell control. Every dot denotes a separate mouse. One-way analysis of variance (ANOVA) was used to examine the data, and Tukey’s multiple comparisons test was used to compare each group’s mean with the means of all other groups. *, *p* < 0.05; **, *p* < 0.01; ***, *p* < 0.001; and ****, *p* < 0.0001.

The indirect ELISA technique was utilized to quantify the titers of specific IgG isotypes (such as IgG1 and IgG2a) in the serum of mice in order to evaluate the variations in humoral immunity induced by immunizing them with tgE537, tgE200, and tgE350. Figure 4A shows the serum IgG antibody titers after the second vaccination. The tgE537-immunized mice showed increased immunogenicity (a titer of IgG antibodies of 64,900), while the tgE350 + Al/CpG group’s IgG antibody titer was 58,533. Like the PBS control group, the tgE200 + Al/CpG-vaccinated group did not show gE-specific IgG antibodies (Figure 4A). The tgE537 + Al/CpG group and the tgE200 + Al/CpG group differed statistically significantly (*p* < 0.001), as did the tgE537 + Al/CpG group and the tgE350 + Al/CpG group (*p* < 0.01) and the tgE537 + Al/CpG group and the PBS group (*p* < 0.001).

The IgG1 and IgG2a antibody titers in serum were also evaluated using the ELISA method to determine the Th1/Th2 balance in humoral immune responses generated by diverse proteins paired with adjuvants. As shown in Figure 4B, the tgE350 + Al/CpG group elicited the highest IgG2a titer (5946), which was significantly different from the PBS group and the tgE200 + Al/CpG group (*p* < 0.01). Notably, while the IgG2a response to tgE350 + Al/CpG was the highest, it did not differ significantly from the response induced by tgE537 + Al/CpG. The IgG2a antibody titer of 5587 in the tgE537 + Al/CpG group was substantially greater compared to the tgE200 + Al/CpG group and the PBS group (*p* < 0.05). The tgE537 + Al/CpG group had the highest IgG1 antibody titer (10,876), as shown in Figure 4C. This group differed considerably from the other three groups (*p* < 0.0001). The tgE200 + Al/CpG group, tgE350 + Al/CpG group, and PBS group had IgG1 antibody titers of 1686, 848, and 50, in that order.

As illustrated in Figure 4D, each dot represents the neutralizing antibody titer of a serum sample from an individual mouse. The tgE350 + Al/CpG group consistently demonstrated superior immunogenicity, generating the highest neutralizing antibody titer of 2718. This group’s performance was significantly superior to all other groups tested. Specifically, the tgE350 + Al/CpG group elicited significantly higher titers compared to the tgE537 + Al/CpG group (*p* < 0.001). The difference was even more pronounced when compared to the tgE200 + Al/CpG group (*p* < 0.0001). Interestingly, while the tgE537 + Al/CpG group induced substantial neutralizing antibody levels, it was less potent than the tgE350 + Al/CpG group, suggesting that the shorter tgE350 construct may be more effective at eliciting neutralizing antibodies.

Titers of neutralizing antibodies are another crucial benchmark for assessing the effectiveness of vaccinations in providing protection. As seen in Figure 4D, each dot represents the neutralizing antibody titer of a serum sample from an individual mouse, and the tgE350 + Al/CpG group continued to generate the highest neutralizing antibody titers (2718), which were considerably greater than those of the tgE537 + Al/CpG group (*p* < 0.001), the tgE200 + Al/CpG group (*p* < 0.0001), and the PBS group (*p* < 0.0001).

**Figure 5:**
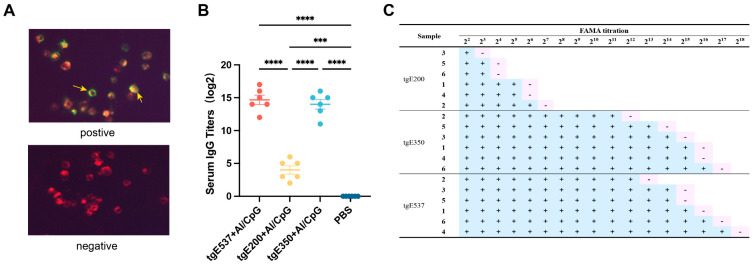
To prepare antigen slides, 2BS cells were infected with VZV. A primary antibody (mouse serum) serially diluted at a constant factor was then added, followed by the addition of a secondary antibody (FITC goat anti-mouse IgG) diluted with 0.01% Evans blue coloring solution. (**A**) Mouse serum samples, both positive and negative; the cell nucleus is shown in red, and antigen–antibody complexes are shown by green fluorescent rings, denoted by yellow arrows. (**B**) Mouse serum IgG antibody levels; (**C**) the serum dilution values of each group’s test samples, where “+” denotes the presence of a complete green fluorescent ring signal and “−” denotes the absence of a complete green fluorescent ring signal or no fluorescent ring signal. N = 6. Every dot denotes a separate mouse. One-way analysis of variance (ANOVA) was used to examine the data, and Tukey’s multiple comparisons test was used to compare each group’s mean with the means of all other groups. ***, *p* < 0.001; and ****, *p* < 0.0001.

This investigation additionally used fluorescent antibody to membrane antigen (FAMA), the gold standard for IgG antibody detection, as ELISA testing can occasionally provide false-positive findings. A complete fluorescent halo indicates a positive sample, whereas an incomplete or absent fluorescent halo around the cell borders indicates a negative sample (Figure 5A). Antibodies in positive samples form antigen–antibody complexes with antigens on the surface of infected cells. The usage of serially diluted sera from mice immunized with various proteins was seen using FAMA staining; the existence of a green fluorescent halo was indicated by a “+”, while the lack of any halo or a full green fluorescent halo was indicated by a “−”. Figure 5C shows an overview of all sample test findings. The IgG antibody titers produced by the tgE350 + Al/CpG and tgE537 + Al/CpG groups were comparable to the ELISA findings and both were higher than those of the tgE200 + Al/CpG group.

## 4. Discussion

There has been an observed rise in the number of herpes zoster (HZ) patients worldwide in the context of the COVID-19 pandemic [15,16]. In Turkey, areas with high incidence rates of COVID-19 have shown an increase in HZ incidence from 0.43% to 0.72% [17]; in Brazil, the HZ cases per million population increased by 10.7% on average across all regions during the same period in 2020 [16]. Despite the lack of conclusive evidence, research has indicated that the COVID-19 vaccine may reactivate the varicella zoster virus (VZV), which is the cause of an increase in HZ cases [18]. According to certain research, vaccination with COVID-19 adenovirus vector vaccines, attenuated live vaccines, or mRNA vaccines increases the prevalence of HZ [19]. Therefore, in order to prevent the reactivation of VZV, some researchers recommend that high-risk groups receive a vaccine against HZ before receiving the COVID-19 vaccine [20].

The live attenuated vaccination (Changchun BCHT Biotechnology Co., Changchun, China) and the recombinant protein vaccine Shingrix (GlaxoSmithKline, GSK) are the two HZ vaccines that are currently approved for sale in China. Compared to the live attenuated vaccine, the recombinant protein vaccine is better suited for patients with primary or secondary immunodeficiencies because it does not involve viral replication. It has also demonstrated strong immunogenicity in patients with underlying conditions like diabetes and chronic obstructive pulmonary disease [19,20,21].

It has been demonstrated that truncated proteins yield good immunogenicity during the creation of recombinant vaccines. For example, the developed Spike protein truncation T1 not only retained the antigenicity of the full-length Spike but also demonstrated an enhanced binding capacity to polyclonal antibodies [22]. Similarly, an adenovirus vaccine containing the truncated S1 protein of SARS-CoV-2 can still induce a strong humoral immune response in mice [23]. The first 1 to 37 amino acids make up the signal peptide, which is retained in truncated proteins because it is essential for directing the release of intracellular proteins into the extracellular environment and increasing protein secretion expression [24,25].

Through examinations of antigen surface accessibility, possible B-cell epitopes, and secondary structure integrity (Figure 1A–D), we analyzed VZV gE. Based on our previous research on VZV gE (1–537AA), we further truncated the protein into two segments: AAs 1–200 and AAs 1–350 (Figure 1E). To evaluate the capacity of the expressed proteins tgE537, tgE200, and tgE350 to induce humoral and cellular immune responses against HZ infection, mice were immunized with them.

Recombinant protein vaccines aim to re-establish humoral and cellular immunity specific to VZV in order to stop the virus from reactivating and spreading to cause herpes zoster (HZ) and its associated consequences. Research has demonstrated an inverse relationship between the strength of cell-mediated immunity and the occurrence and severity of HZ infections, suggesting that a reduction in cellular immunity could exacerbate HZ infections. Thus, cellular immunity is essential for the protective effects of HZ vaccines [26]. We examined the number of splenocytes generating IFN-γ and IL-4 using ELISPOT assays (Figure 3A–C) and evaluated the distribution of various cell types among splenocytes using flow cytometry (Figure 3D–H). The tgE200 + Al/CpG and tgE350 + Al/CpG groups were able to elicit cellular immune responses akin to those of the tgE537 + Al/CpG group. Since the immunogenicity of recombinant vaccines is lower than that of live attenuated vaccines, cytotoxic cells are produced in the body, but most infiltrating CD4^+^ and CD8^+^ T cells lack significant cytotoxic cell markers [27]; conversely, the immune response is below the detection level of immunohistochemical assays, and so no differences in lymphocyte numbers were observed [28]. The idea that live attenuated vaccines are the best immunogens appears to be contradicted by certain research that indicates that recombinant HZ vaccines are more immunogenic than live attenuated vaccines. Adjuvants, however, can greatly increase the immunogenicity of subunit vaccines, making up for the robust T- and B-cell responses brought on by active viral replication, which explains why there is a discrepancy [13,29]. Although the traditional adjuvant Al/CpG was utilized in this trial, other factors, such as the vaccination dosage and type, may have greater effects on the immune system.

While cell-mediated immune responses are essential in stopping the varicella zoster virus (VZV) from reactivating, antibodies that can efficiently remove infected cells are also essential [30]. Antibodies specific to VZV are important in the removal of the reactivated virus. The significance of IgG antibodies in immunological processes, including phagocytosis and antigen neutralization, is shown by the quick rise in the creation and application of IgG-based treatments [31]. As seen in Figure 4A–C, both the tgE350 + Al/CpG and tgE537 + Al/CpG groups were able to create particular protective antibodies in contrast to the truncated tgE200. The FAMA approach is the gold standard for VZV antibody detection in serum, and the results in Figure 5C corroborate the accuracy of the preceding conclusion [32]. By attaching to viral particles, antibodies exposed to viral surface antigen epitopes can stop cellular infection. This kind of viral neutralization is especially efficient against reinfection [33]. When used in conjunction with the truncated protein NΔ21 of interferon-induced transmembrane protein 3 (IFITM3), the inactivated influenza vaccine can produce high titers of neutralizing antibodies in mice [34]. Similarly, choosing the RBD region of the truncated S protein antigens of SARS-CoV-2 can produce higher antibody titers [35]. In terms of clinical effectiveness, the majority of protective vaccines rely on neutralizing antibodies [36]. VZV gE’s affinity enhancement has a strong correlation with the neutralizing antibody enhancement, suggesting that gE is a crucial target for neutralizing antibodies [37]. The generation of large titers of neutralizing antibodies following vaccination is a reliable sign of vaccine protection. The antibodies generated by the HZ vaccine should be capable of neutralizing virus particles. The tgE350 group had the greatest neutralizing antibody titers (Figure 4D,E). In this experiment, mice were immunized with three different lengths of the VZV gE protein. Both the tgE350 + Al/CpG and tgE537 + Al/CpG groups were able to induce neutralizing antibodies, with tgE350 unexpectedly producing higher titers. This could be related to the glycosylation pattern of these proteins. While some studies suggest that O-linked glycans can enhance antibody binding [13,38], the specific role of glycosylation in our observed results requires further investigation. Alternatively, it could be because immunodominant non-neutralizing epitopes within VZV gE can trigger an immune response to the host, while neutralizing epitopes remain unresponsive [36]. We deduce that tgE350 contains more potent immunogenic epitopes in the area between amino acids 200 and 350 (Figure 1B). Previous studies using Western blotting and ELISAs of sera from herpes zoster and chickenpox patients have identified the immunodominant regions of gE as amino acids 1–134 and 101–161 [39]. In contrast, our experimental results in mice indicate that the region comprising amino acids 200–350 of gE exhibits stronger immunogenic effects. This discrepancy suggests that this area warrants further investigation. However, it is important to note that our findings are based on a murine model and may not directly translate to human immune responses. The observed differences could be due to species-specific immune responses or methodological variations. Further studies, particularly in human cell systems or humanized mouse models, are necessary to validate these results and explore their relevance to human VZV immunity. Remarkably, vaccination occasionally produces weak or non-neutralizing antibodies, which bind to the virus and cell surface Fc receptors through their Fc regions, facilitating viral entry into cells and potentially increasing viral virulence and infectivity. This phenomenon is referred to as antibody-dependent enhancement (ADE) of infection. COVID-19, dengue, and respiratory syncytial virus (RSV) vaccinations have all been linked to this side effect. Therefore, more research in independent trials is necessary to confirm whether the high titers of neutralizing antibodies produced by the tgE350 vaccinated group in this study might cause ADE and affect the safety of the truncated vaccines.

The live attenuated vaccine containing the VZV-Oka strain is commonly used and is very efficient at fighting HZ illness. Live attenuated vaccines, however, run the danger of switching back to more virulent forms. The significant prevalence of HZ, particularly in underdeveloped areas, makes the creation of a more affordable and safe vaccines extremely important. Recombinant protein vaccines show a lot of promise due to their quick development cycle, low cost, and good safety profile [23]. Three distinct lengths of the VZV glycoprotein E (gE)—tgE537, tgE200, and tgE350—were effectively produced in vitro for this work, and their immunogenicity was evaluated. The findings demonstrated that the tgE350 and tgE537 proteins elicited considerably larger titers of neutralizing antibodies and strong humoral immune responses. Since large titers of neutralizing antibodies are essential to the effectiveness of varicella vaccinations, our results also shed light on the development of such vaccines [40]. This study proposes novel approaches for the creation of vaccines against different herpesviruses, in addition to providing fresh insights into the production of truncated protein vaccines based on VZV gE.

This study, while yielding promising results, has two main limitations that warrant attention in future research. Firstly, the use of PBS alone as a negative control may have restricted our ability to fully assess adjuvant-induced non-specific immune responses, necessitating the inclusion of both PBS and adjuvant-only control groups in future studies to better differentiate vaccine-specific immune responses from potential adjuvant-induced effects. Secondly, the lack of long-term immune response data is a significant limitation; while we observed robust initial responses, their durability remains unknown, highlighting the need for longitudinal tracking of antibody titers and memory cell responses in future investigations. Addressing these limitations in subsequent studies will provide a more comprehensive understanding of our candidate vaccine’s efficacy and longevity, thereby informing potential clinical applications and future vaccine development strategies.

## 5. Conclusions

In this study, three truncated variants of the varicella zoster virus (VZV) glycoprotein E (gE) with varying lengths, namely, tgE537, tgE200, and tgE350, were designed through bioinformatics analysis. These proteins were expressed, purified, and then used to immunize mice in combination with the Al/CpG adjuvant to evaluate their ability to induce humoral and cellular immune responses. The results demonstrated that both tgE537 and tgE350 were capable of eliciting VZV-specific antibodies and neutralizing antibodies, with tgE350 exhibiting the highest neutralizing antibody titer (4388). In contrast, tgE200 failed to trigger an equivalent humoral immune response. Collectively, these findings suggest that tgE350 is a highly promising truncated protein antigen that lays the foundation for the development of more effective and safer recombinant protein vaccines against herpes zoster. However, further investigations are warranted to assess its long-term immunoprotective efficacy and potential adverse effects.

## Figures and Tables

**Table 1:** Mouse immunization strategy.

Vaccine Group	Ag (μg)	Adj (μg)	Mice	Injection
1. tgE537 + Al/CpG	5 μg	50 μg Al + 10 μg CpG	6	I.M.
2. tgE200 + Al/CpG	5 μg	50 μg Al + 10 μg CpG	6	I.M.
3. Tg350 + Al/CpG	5 μg	50 μg Al + 10 μg CpG	6	I.M.
4. PBS	-	-	6	I.M.

- Not added; I.M., intramuscular injection.

## Data Availability

All the data from the study are available from the corresponding author upon reasonable request.
